# Nk3R blockade has sex-divergent effects on memory in mice

**DOI:** 10.1186/s13293-022-00437-z

**Published:** 2022-06-11

**Authors:** Antonio Florido, Estefanía Moreno, Enric I. Canela, Raül Andero

**Affiliations:** 1 Institut de Neurociències, Universitat Autònoma de Barcelona, Cerdanyola del Vallès, 08193 Barcelona, Spain; 2 Departament de Psicobiologia i Metodologia de les Ciències de la Salut, Universitat Autònoma de Barcelona, Cerdanyola del Vallès, 08193 Barcelona, Spain; 3 Departament de Bioquímica i Biomedicina Molecular, Facultat de Biologia, Universitat de Barcelona i Institut de Biomedicina de la Universitat de Barcelona (IBUB), 08028 Barcelona, Spain; 4 Instituto de Salud Carlos III, 28029 Madrid, Spain; 5 Centro de Investigación Biomédica en Red en Salud Mental (CIBERSAM), Instituto de Salud Carlos III, 28029 Madrid, Spain; 6 Unitat de Neurociència Translacional, Parc Taulí Hospital Universitari, Institut d’Investigació I Innovació Parc Taulí (I3PT), Universitat Autònoma de Barcelona, Cerdanyola del Vallès, 08193 Barcelona, Spain; 7 ICREA, Pg. Lluís Companys 23, 08010 Barcelona, Spain

**Keywords:** Neurokinin-3 receptor, Osanetant, Sex differences, Heterodimerization, Estradiol, Testosterone

## Abstract

**Background:**

Memory consolidation is a process required for the formation of long-term memories. The G-protein-coupled receptor (GPCR) neurokinin-3-receptor (Nk3R) and its interactions with sex hormones seem important for the modulation of fear memory consolidation: Nk3R antagonism in male mice impairs fear memory, but enhances it in females. However, the involvement of the Nk3R as a modulator of other memories in both sexes remains unexplored.

**Methods:**

We use the novel object recognition paradigm to test the effect of a systemic blockade of Nk3R during memory consolidation. Further, we assess the expression of estrogen receptor α, estrogen receptor β, and androgen receptor and heterodimerization with Nk3R in the medial prefrontal cortex (mPFC) and dorsal hippocampus (DH) of mice.

**Results:**

Nk3R systemic antagonism elicited decreased memory consolidation in males while it enhanced it in females during proestrus. Nk3R analysis in the different subregions of the mPFC and the DH showed a higher expression in males than females. Moreover, females presented upregulation of the androgen receptor in the CA1 and the estrogen receptor beta in the cingulate cortex, CA1, and dentate gyrus. Overall, males presented an upregulation of the estrogen receptor alpha. We also explored the heterodimerization of GCPR membrane sex hormone receptors with the Nk3R. We found a higher percentage of Nk3R-membrane G-protein estrogen receptors heterodimers in the prelimbic cortex of the mPFC in females, suggesting an interaction of estradiol with Nk3R in memory consolidation. However, males presented a higher percentage of Nk3R-membrane G-protein androgen receptors heterodimers compared to females, pointing to an interaction of testosterone with Nk3R in memory consolidation.

**Conclusion:**

These data propose novel ideas on functional interactions between Nk3R, sex hormones, estrogen receptors, and androgen receptors in memory consolidation.

## Highlights

- Nk3R antagonism reduces recognition memory consolidation in male mice and increases it in proestrus females.
- Androgen receptor expression is higher in the CA1 compared to DG, CA3, and the mPFC.
- Estrogen repcetor α expression is higher in males than in females in the DH and mPFC.
- Estrogen receptor β expression is greater in females than in males in the DG, CA1, and CG.
- Over 60% of Nk3R in the DH and mPFC is heterodimerized with membrane estrogen receptor and androgen receptor.
- Nk3R–GPAR is more abundant in males than in proestrus females, whereas Nk3R–GPER is greater in proestrus females compared to males.

## Background

Recognition memory is a type of declarative memory that allows mammals to identify an object or situation as familiar, in contrast with novel objects or situations. The organism uses this memory to compare novel situations with past personal experiences generating a congruent response toward the novel situation [1]. Although rodents do not present declarative memory itself, since they do not have the ability to explicitly expose information, analogous procedures have been developed to test this type of memory in rats and mice [2]. Declarative memory in humans has usually been related to spatial and recognition memory in mice [3, 4], and in both cases, it has been established common fundamental encoding processes in the hippocampus and its connections [5]. Memory consolidation represents a crucial point in this process when recently labile short-term memories are transformed into long-term and stable memory traces [2]. This transformation typically requires cellular and molecular adaptations, such as genetic regulation of immediate-early gene expression or increased density of dendritic spines [1]. The tachykinin 2 (Tac2) pathway is characterized by the synthesis of neurokinin B (NkB) from the Tac2 gene and its binding to the neurokinin 3 receptor (Nk3R) mediating sex differences in the consolidation of fear memories [4, 6, 7]. Moreover, Nk3R manipulations in male rodents have been shown to modulate the formation of hippocampus-dependent declarative memory [8, 9]. Interestingly, Nk3R antagonists are safe and well-tolerated drugs in humans [10] which makes them potentially attractive candidates for treating mental disorders with memory alterations. Osanetant, a potent selective Nk3R antagonist, showed safety in a clinical trial for the treatment of psychotic and affective symptoms typical of schizophrenia [11]. Although this study was discontinued, Nk3R antagonists are currently under study as a promising solution for induced vasomotor symptoms (iVS) during menopause [12]. Surprisingly, there is only one prior study that has included females involving the Tac2 pathway and memory formation [7]. The abovementioned study shows how Nk3R blockade, as well as Tac2 silencing in the amygdala, reduce fear memory consolidation in male mice while increasing it in females [7]. In contrast, the role of the amygdala in recognition memory is limited and appears to be circumscribed to the familiarization phase, but not recall [13]. On the other hand, the hippocampus and the medial prefrontal cortex (mPFC) play a major role in the consolidation of recognition memory in mice [14, 15]. Hippocampal lesions after familiarization with two objects have been shown to disrupt object recognition in male rats [16]. Moreover, the dorsal hippocampus (DH) is highly interconnected with the mPFC [17, 18]. This network has been demonstrated to mediate whether an object was previously encountered or not [19]. Moreover, lesions of the prelimbic cortex (PrL) and infralimbic cortex (IL) of the mPFC produce deficits in recognition memory [20, 21]. These findings support the role of the mPFC–DH connections in the formation of recognition memory.

The Nk3R has been shown to interact with sex hormones in both males and females in the modulation of fear memory consolidation through the modulation of the intracellular cascade comprising Akt/GSK3β/β-Catenin [7]. In female mice, estradiol enhances recognition memory consolidation, via the activation of the PI3K/Akt pathway in the DH [22] in a similar manner. In addition, testosterone in male rats has been shown to enhance synaptic plasticity in the dentate gyrus and increase reference memory measured by the inspection of non-baited holes in a hole board [23].

In recent years, a substantial amount of evidence has shown that G-protein-coupled receptors (GPCRs) may exist as dimeric or oligomeric complexes, exhibiting biochemical properties distinct from those of the protomers in modifying cellular responses [24]. Nk3R couples to the pertussis toxin-insensitive G-protein Gq, whose activation results in the production of inositol triphosphate and diacylglycerol, activating intracellular signaling cascades that result in memory modulation [6, 25]. Notwithstanding, heterodimerization of Nk3R with other GPCRs remains unexplored.

Nk3R antagonists have shown sex-dependent effects on fear memory and sex hormonal fluctuations [7, 26], but sex differences in recognition memory after Nk3R antagonism have not been tested yet. Here, we found that systemic Nk3R antagonism modulated recognition memory consolidation in an opposite-sex manner, reducing consolidation in males and increasing it in females during proestrus (high levels of estradiol). Then we measured the Nk3R, estrogen receptors α (ERα) and β (ERβ), and androgen receptors (AR) in the DH and mPFC uncovering sex differences. Finally, we studied protein interactions between the Nk3R and membrane G-protein estrogen receptors (GPER) and G-protein androgen receptors (GPAR) measuring heterodimerization as a possible mechanism of the sex differences in memory formation upon Nk3R antagonism.

## Methods

### Animals

All experiments used male and naturally cycling female C57BL/6J adult mice (Charles River, Barcelona, Spain), 8 weeks old at the beginning of the experiments and housed in groups of 4 in a room with a 12:12 h light/dark cycle (lights on from 8 am to 8 pm). All animals were housed with a controlled temperature of 22 ± 1 °C and humidity (~ 40%). Behavioral procedures and pharmacological manipulations began early in the light phase of the cycle. Male and female mice were housed separately in the same room. Experiments in mice were performed under the approved ethics protocol with reference CEEAH 3603. All procedures were approved by the Ethics Committee of the Universitat Autònoma de Barcelona and the Generalitat de Catalunya. Experiments were also carried out following the European Communities Council Directive (2010-63-UE) and Spanish legislation (RD 53/2013).

### Drugs

Osanetant was purchased from Merck (SML0798) and prepared for 5 and 10 mg/kg. The vehicle used for this preparation was 0.1% (v/v) Tween 20 in 0.9% (m/v) NaCl solution. Osanetant and its vehicle were administered at 10 mL/kg 30 min after the familiarization phase of the novel object recognition (NOR), to isolate the effect of the drug on the consolidation window of familiarization consolidation. Given previous data that support an attenuated effect of osanetant in memory during estrus, metestrus, and diestrus [7]; only females during the proestrus stage of the estrous cycle during familiarization were used in this experiment.

### Determination of the estrous cycle

Determination of the estrous stage in females was performed by assessing vaginal smear cytology [27]. To assess the phase of the estrous cycle that females presented during the familiarization phase of the NOR, all-female mice were monitored for 3–4 consecutive cycles (approximately 10–14 days) before familiarization to test for regularity of the cycle. We performed a vaginal lavage with a 20-μL pipette that was loaded with 10 μL of standard NaCl 0.9% (w/v) solution, and later the tip was softly placed on the vaginal aperture. Before the collection of the sample, urine was softly removed using a tissue. The 10 μL of the sample were unloaded and collected 5 consecutive times to collect enough cells for the assessment, and later placed on an adhesion slide (Superfrost Plus, Thermo Fisher). All vaginal smear samples were collected between 9 and 10 am. Slides were dried using a hot plate (HI1220, Leica) at 37 °C for 30 min before staining in cresyl violet acetate (C5042, Merck) 0.1% (v/v) for 5 min, washed twice for 1 min in distilled water, and read in brightfield microscopy with a 10× or 20× objective in an Eclipse 80i microscope (Zeiss, Spain).

Three different cell types may appear in the preparation: cornified epithelial cells, round nucleated epithelial cells, or leukocytes. The different stages of the estrous cycle were assessed depending on the proportion of the abovementioned cells. Proestrus is characterized by a high proportion (> 80%) of nucleated epithelial cells that might present very small amounts of cornified epithelial cells or leukocytes. Estrus is typically presented with cornified epithelial cells with a lower grade of staining than leukocytes and nucleated epithelial cells. Metestrus presents a mixture of cornified epithelial cells and a considerable proportion of leukocytes. Diestrus is characterized by > 90% of leukocytes that might present a very small proportion of round nucleated epithelial cells [27].

### Novel object recognition

NOR is widely accepted as a test to assess prefrontal and hippocampal-dependent learning and memory processes [28, 29]. Before starting the NOR test, a different cohort of animals was used to test that animals did not present differences in the time exploring each object of the NOR test.

All animals were habituated to the testing arena for 5 min for 2 consecutive days before the familiarization phase of the test. On the 3rd day, animals were placed in the arena where they freely explored two identical objects (either a yellow plastic pin or a purple crystal jar) for 15 min. Male and female mice received 5 mg/kg, 10 mg/kg osanetant, or vehicle 30 min after the familiarization phase of the NOR test before returning to their homecage. The percentage of time exploring each object was calculated as a measure of preference for any of two identical objects.

**Fig. 1:**
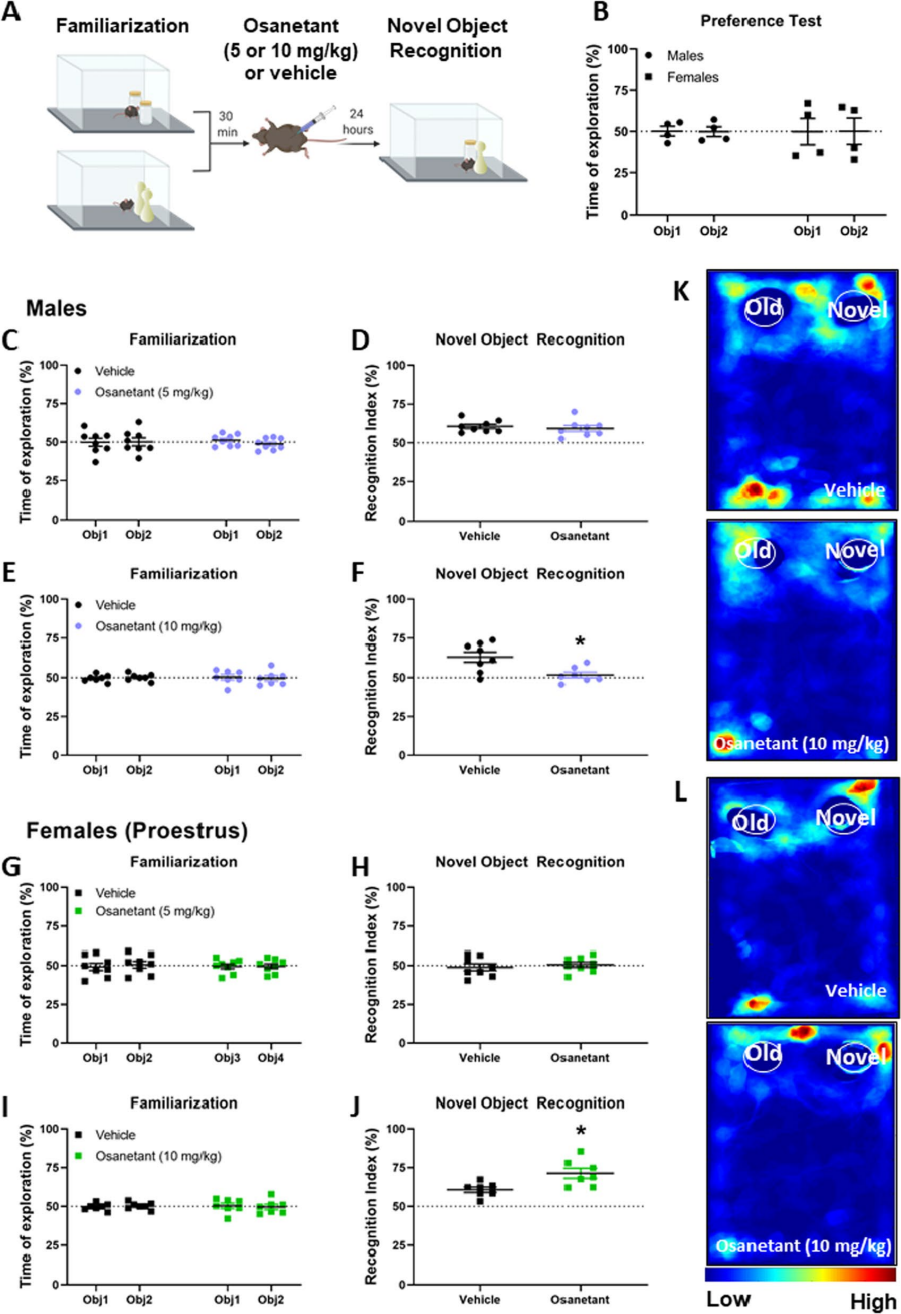
Novel object recognition (NOR) test after the administration of osanetant or vehicle. **A** Schematic representation of the behavioral procedure. **B** Males and females’ preference test between the two objects used in the test. **C**, **E** Familiarization phase of the NOR task in males before receiving osanetant (5 or 10 mg/kg) or vehicle. **D** NOR test after receiving 5 mg/kg or vehicle after familiarization in males. **F** NOR test after receiving 10 mg/kg or vehicle after familiarization in males. **G**, **I** Familiarization phase of the NOR task in females (proestrus) before receiving osanetant (5 or 10 mg/kg) or vehicle. **H** NOR test after receiving 5 mg/kg or vehicle after familiarization in females (proestrus). **J** NOR test after receiving 10 mg/kg or vehicle after familiarization in females (proestrus). The recognition index is calculated by dividing the time exploring the novel object by the total time of exploration and expressed as a percentage. The dashed line in each graph represents 50% of the relative exploration time. **K** Representative heatmaps of the NOR test showing males that received vehicle or osanetant (10 mg/kg). **L** Representative heatmaps of the NOR test showing females (proestrus) that received vehicle or osanetant (10 mg/kg). Data were analyzed using Three-way ANOVA for the familiarization phase (Sex × Object × Treatment) or Two-way ANOVA (Sex × Treatment). *p < 0.05. Obj1 = Object 1; Obj2 = Object 2

On the test day, the animals returned to the arena where they freely explored the two objects for 15 min. The novel object was the yellow plastic pin for half of the animals and the purple crystal jar for the other half. Further, we counterbalanced the position of the novel object (right or left) to avoid an effect of place preference. All phases of the test were carried out under ~ 15 lux. The relative time exploring the novel object, expressed in percentage, was calculated as a measure of recognition by an experienced observer using a stopwatch. Heatmaps were obtained using Autotype for MATLAB (Fig. 1K, L).

### Immunofluorescent determination of Nk3R, ERα, ERβ, and AR

Immunohistochemical assays were performed on naïve males and proestrus females whose tissue was collected early in the morning by transcardial perfusion with 50 mL of 4% (m/v) of buffered paraformaldehyde (PFA) (Casa Álvarez, Barcelona, Spain). These animals were naïve and had not undergone any treatment or behavioral procedure before tissue collection. Brains were stored in the same solution at 4 °C for postfixation overnight before being transferred to 30% (m/v) sucrose in Sorensen’s PB buffer for equilibration in conic Falcon tubes. After 48 h of equilibration in the sucrose solution, brains were snap-frozen in chilled isopentane and stored at − 80 °C until sectioning.

Brains were coronally sectioned at 30 um/slice using a Leica cryostat (− 20 °C for the chamber, − 18 °C for the sample) and stored in cryoprotective solution before staining. For staining, brain slices containing the mPFC and DH were rinsed in KPBS 1× three times for 10 min each, before spending 1 h in incubation in blocking buffer (5% normal donkey serum in 0.4% Triton X solution prepared with KPBS 1×), to avoid nonspecific binding of the primary antibody. Then, slices were incubated overnight at 4 °C in 300 µL of blocking buffer containing either one of the following combinations: rabbit anti-Nk3R (1:1500, donated by Phillipe Cioffi) and mouse monoclonal anti-ERa (1:50, sc-8002, Santa Cruz Biotechnology), rabbit anti-Nk3R and mouse monoclonal anti-ERb (1:500, ab288, Abcam) or rabbit anti-Nk3R and mouse monoclonal anti-AR (1:50, sc-7305, Santa Cruz Biotechnology). After primary antibody incubation, brain slices were rinsed three times, 10 min each, with KPBS 1× and incubated at room temperature for 2 h in blocking buffer containing donkey anti-rabbit AlexaFluor488 (1:1000, 115-546-072, Jackson Immunoresearch) and donkey anti-mouse Rhodamine Red (1:1000, 715-295-150, Jackson Immunoresearch). Slices were then rinsed again and incubated for 3 min with 4′,6-diamidino-2-phenylindole (DAPI) (1:10,000, 10236276001, Merck) to stain cell nuclei before mounting and covering.

Z-Stacks of the cingulate cortex (CG), PrL, IL, DG, CA1, and CA3 (50 μm/interval) were acquired using a Leica SP3 confocal microscope with a PL APO 40× immersion objective, controlled by Zen 2010 software. The integrated density of the signal was then calculated using Fiji for Windows v1.52.n and used as a measure of the protein quantity of the taken pictures.

### In situ proximity ligand assays (PLA)

With PLA, primary antibodies raised in different species are used to detect different proteins. Secondary antibodies specific to each primary antibody that include a short sequence of DNA are used to detect the presence of the primary antibodies. If both primary antibodies are part of the same protein complex, both DNA chains react and, after amplification of this DNA, fluorescent complementary oligonucleotides are used to detect the heterodimers. Naïve mice were deeply anesthetized and immediately perfused transcardially with buffered 4% paraformaldehyde (Casa Álvarez, Barcelona, Spain). Brains were removed and post-fixed overnight in the same solution, cryoprotected by immersion of 30% sucrose in Sorensen’s PB buffer for 48 h at 4 °C, and then snap-frozen in dry ice-cooled isopentane. Serial coronal cryostat sections (30 μm) through the whole brain were collected in a cryoprotective solution and stored at − 20 °C until PLA experiments were performed.

Immediately before the assay, mouse brain sections were mounted on glass slides, washed in PBS, permeabilized with PBS containing 0.01% Triton X-100 for 10 min, and successively washed with PBS. Interactions were detected using the Duolink in situ PLA detection Kit (Sigma-Aldrich, St. Louis, MO, USA) following the supplier’s instructions. A mixture of the primary antibodies rabbit anti-Nk3R antibody (1:100; donated by Phillipe Cioffi (INSERM)) directly linked to a plus PLA probe and rabbit anti-GPER antibody (1:100; Sigma; cat HPA027052) directly linked to a minus PLA probe were used to detect Nk3R–GPER interaction, or rabbit anti-GPAR antibody (1:100; Thermo Scientific, Fremont, CA, USA; Cat PA5-21074) directly linked to a minus PLA probe were used to detect Nk3R–GPAR interaction. For negative controls, one of the primary antibodies (anti-GPER or anti-GPAR) was omitted (data not shown). Then, samples were processed for ligation and amplification with a Detection Reagent Red and were mounted using a DAPI-containing mounting medium.

Samples were analyzed in a Leica SP2 confocal microscope (Leica Microsystems, Mannheim, Germany) equipped with an apochromatic 63× oil-immersion objective (1.4 numerical aperture), and 405 nm and 561 nm laser lines. For each field of view, a stack of two channels (one per staining) and 9 to 13 Z-stacks with a step size of 1 µm was acquired. Images were opened and processed with Image J software (National Institutes of Health, Bethesda, MD). Quantification of cells containing one or more red dots versus total cells (blue nuclei) was determined by using the Fiji package (http://pacific.mpi-cbg.de/), considering a total of 600–800 cells from 5 to 10 different fields (63×) within each region from three different animals. Nuclei and red dots were counted on the maximum projections of each image stack. After getting the projection, each channel was processed individually. The blue nuclei and red dots were segmented by filtering with a median filter, subtracting the background, enhancing the contrast with the Contrast Limited Adaptive Histogram Equalization (CLAHE) plug-in, and finally applying a threshold to obtain the binary image and the regions of interest (ROIs). Red spots were counted in each of the ROIs obtained in the nuclei images.

### Experimental design and statistical analyses

The NOR test was analyzed using a multifactorial design Sex × Treatment (2 × 2) and the percentage of exploration of the new object as the dependent variable. The familiarization phase was analyzed using a multifactorial design Sex × Object × Treatment (2 × 2 × 2). Immunohistochemistry assays included main factors Sex and Area (2 × 6) to assess statistical differences or interactions of integrated density between groups on Nk3R, ERα, ERβ, and AR expression in the mPFC and the DH. Heterodimerization analysis included Area and Sex as main factors (6 × 2), and the percentage of cells including dimers among Nk3R positive cells was used as a dependent variable across the mPFC and the DH. The result of all statistical tests is summarized in Table 2.

All statistical analyses were performed using IBM SPSS v23.0. Outliers were detected using Grubb’s test and removed when it was appropriate. Before proceeding with the null hypothesis testing, all datasets were tested for normality and homoscedasticity using the Shapiro–Wilk test and Levene’s test, respectively. Univariate ANOVAs were performed to assess the statistical significance of main effects and interactions, and significant interactions between main factors were analyzed using Bonferroni correction. Results are presented as mean $\pm$ SEM, and statistical significance was set at *p* < 0.05. All graphs presented in the figures were designed using Prism 7 (CA, USA).

## Results

### Nk3R blockade decreases recognition memory consolidation in males and increases it in females during proestrus in a dose-dependent manner

After assessing that animals presented no preference for any of the objects used in the test (Sex: [*F*(1,24) = 0.057, *p* = 0.813]; Object: [*F*(1,24) = 0.072, *p* = 0.791]; Sex × Object: [*F*(1,24) = 0.072, *p* = 0.791] (Fig. 1B), a different cohort of male and female mice underwent the familiarization phase of the object recognition. As expected, familiarization testing showed no differences in the time exploring each object either before receiving osanetant 5 mg/kg (Sex: [*F*(1,56) = 0.000, *p* = 1.000]; Object: [*F*(1,56) = 0.031, *p* = 0.860]; Treatment: [*F*(1,56) = 0.000, *p* = 1.000], Sex × Object: [*F*(1,56) = 0.359, *p* = 0.552], Sex × Treatment: [*F*(1,56) = 0.000, *p* = 0.000], Object × Treatment: [*F*(1,56) = 1.128, *p* = 0.293]; Sex × Object × Treatment: [*F*(1,56) = 0.006, *p* = 0.939]) or 10 mg/kg (Sex: [*F*(1,50) = 0.000, *p* = 1.000]; Object: [*F*(1,50) = 0.000, *p* = 1.000; Treatment: [*F*(1,50) = 0.103, *p* = 0.749]; Sex × Object: [*F*(1,50) = 0.091, *p* = 0.764]; Sex × Treatment: [*F*(1,50) = 0.000, *p* = 1.000]; Object × Treatment: [*F*(1,50) = 1.638, *p* = 0.206]; Sex × Object × Treatment: *F*(1,50) = 2.651, *p* = 0.110]) (Fig. 1C, E).

The recognition test, performed 24 h after receiving the drug, revealed no differences in recognition between males and females (no estrous cycle monitorization) that received 5 mg/kg (Sex: [*F*(1,28) = 1.748, *p* = 0.197; Treatment: [*F*(1,28) = 0.227, *p* = 0.603; Sex × Treatment: [*F*(1,28) = 0.002, *p* = 0.965) (Fig. 1D, H). Given that high levels of circulating estradiol have shown to be necessary for osanetant to modulate memory consolidation in a cued-fear conditioning task [7], we then tested females that were in the proestrus stage of the estrous cycle during the familiarization phase and the administration of the drug. In a different cohort of mice, males that received 10 mg/kg after the familiarization phase presented reduced memory consolidation compared to vehicle administered males, while proestrus females that received 10 mg/kg showed increased performance in the recognition test compared to the vehicle control group (Sex: [*F*(1,25) = 10.595, *p* = 0.003]; Treatment: [*F*(1,25) = 0.022, *p* = 0.884]; Sex × Treatment: [*F*(1,25) = 17.128, *p* = 0.000; treatment in males: [*F*(1,25) = 9.496, *p* = 0.005], and in proestrus females: [*F*(1,25) = 7.714, *p* = 0.010]) (Fig. 1F, J).

This study extends our knowledge on Nk3R antagonism showing that it does not only exert an opposite-sex regulation of memory consolidation in fear memories as it has previously been demonstrated, but also in object recognition.

### Nk3 and AR expression are higher in males compared to females (proestrus), while ERα and ERβ expression depend on sex and area

Our previous research suggests the necessity of adult concentration of sex hormones for osanetant to modulate fear memory consolidation [7]. Following up on this data, we measured the expression of sex hormone receptors (androgen receptor -AR-, estrogen receptor alpha -ERα-, and estrogen receptor beta -ERβ-) in areas related to NOR memory consolidation where the Nk3R might be exerting a regulating function.

**Fig. 2:**
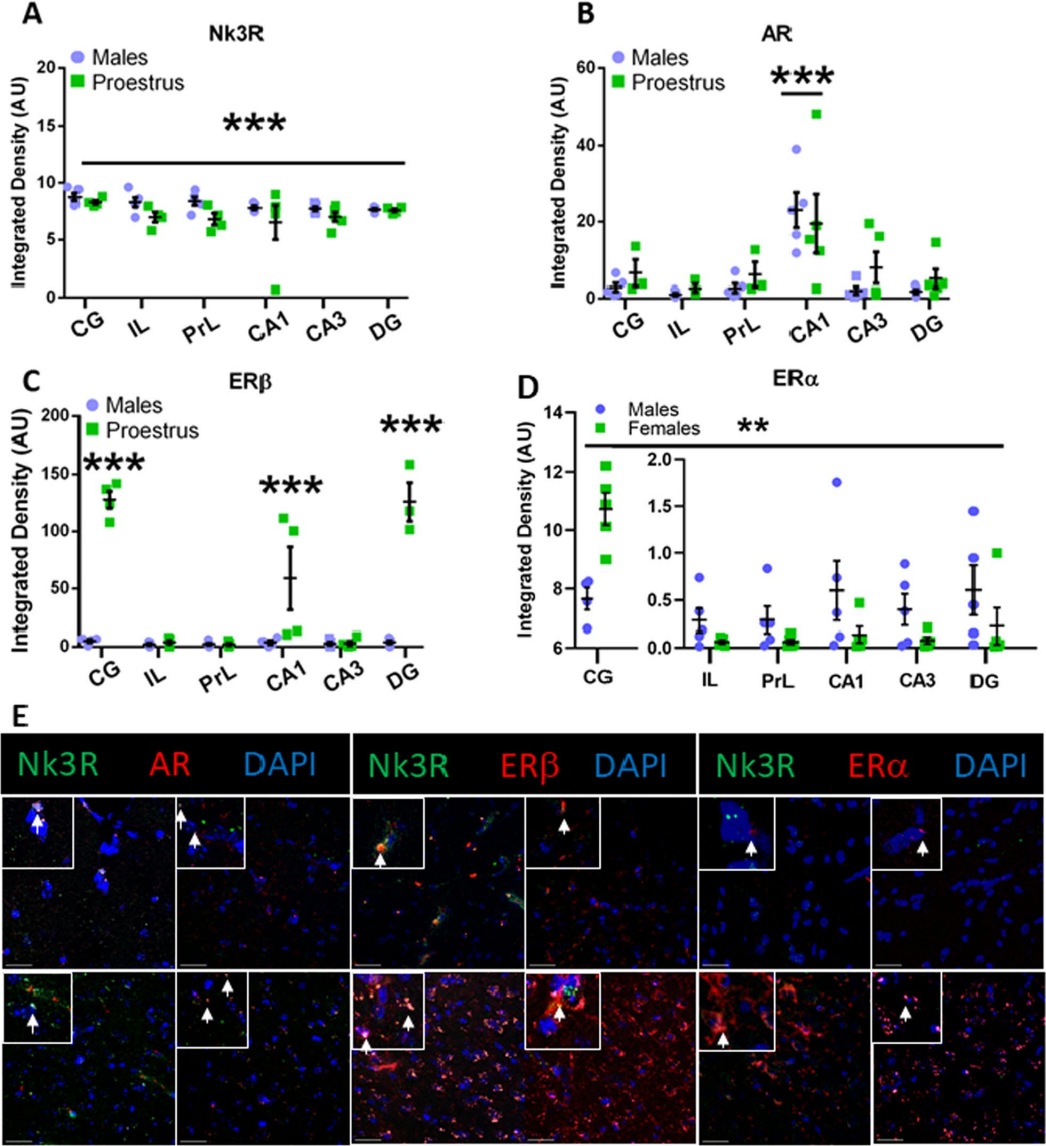
Immunolocalization of neurokinin 3 receptor (Nk3R) with androgen receptor (AR), estrogen receptor α (ERα), and estrogen receptor β (ERβ). **A** Immunolocalization of Nk3R in the medial prefrontal cortex (mPFC) or the dorsal hippocampus (DH). **B** Immunolocalization of AR in the mPFC or the DH. **C** Immunolocalization of ERβ in the mPFC or the DH. **D** Immunolocalization of ERα in the mPFC or the DH. **D** Representative confocal images of AR, ERβ, and ERα with Nk3R in the CG and CA1. *CG* cingulate cortex, *IL* infralimbic cortex, *PrL* prelimbic cortex, *CA1* field CA1 of hippocampus, *CA3* field CA3 of hippocampus, *DG* dentate gyrus. Data are mean ± SEM. Yellow pixels represent colocalization between red and green. White pixels represent the colocalization of red, green, and blue. Datasets were analyzed using two-way ANOVA. **p < 0.01, ***p < 0.001. Asterisks above a line in **A** and **D** indicate a significant main effect of sex independently of the area. Asterisks in **B** and **C** indicate significant main effect sex in discrete areas after finding a significant Sex × Area interaction. **E** Yellow pixels indicate colocalization between Nk3R and AR, ERβ, or ERα. White pixels indicate colocalization between DAPI, Nk3R, and AR, ERβ, or ERα. Scale bar = 25 µm

To understand a possible role for Nk3R and sex hormones in memory consolidation, we performed immunolocalization assay to assess the expression of Nk3R, ERα, ERβ and AR in the DH (CA1, CA3 and DG) and the mPFC (CG, PrL and IL), areas that are typically related to the consolidation of recognition memory. This analysis revealed an overall increased expression of Nk3R in males compared to females in the DH and the mPFC (Sex: [*F*(1,43) = 7.770, *p* = 0.008]; Area: [*F*(5,44) = 1.631, *p* = 0.257]; Sex × Area: [*F*(5,44) = 0.768, *p* = 0.758]) (Fig. 2A, E). The expression of ERα was also enhanced in male mice compared to proestrus female mice in the DH and the mPFC (Sex: [*F*(1, 47) = 7.001, *p* = 0.011]; Area [*F*(5,47) = 1.789, *p* = 0.133]; Sex × Area: [*F*(5,47) = 1.326, *p* = 0.270]) (Fig. 2D, E). Contrarily, the expression of ERβ was greater in proestrus females compared to males in CA1, DG and CG (Sex: [*F*(1,41) = 115.275, *p* = 0.000]; Area [*F*(5,41) = 28.398, *p* = 0.000]; Sex × Area [*F*(5,41) = 26.475, *p* = 0.000], CA1 [*F*(1,41) = 21.410, *p* = 0.000], CA3 [*F*(1,41) = 0.001, *p* = 0.973], DG [*F*(1,41) = 98.405, *p* = 0.000], CG [*F*(1,41) = 105.908, *p* = 0.000], PrL [*F*(1,41) = 0.029, *p* = 0.866], IL [*F*(1,41) = 0.000, *p* = 0.985]) (Fig. 2C, E). Interestingly, no differences between areas or sexes were found in the analyses AR expression in the DH and mPFC of male and proestrus females, but a sex-independent increase of AR expression (Sex: [*F*(1,42) = 1.388, *p* = 0.245]; Area [*F*(5,42) = 9.144, *p* = 0.000]; Sex × Area [*F*(5,41) = 0.475, *p* = 0.793]; CG vs rest of the areas *p* = 0.000) (Fig. 2B, E).

### Heterodimerization of Nk3R with the membrane estrogen receptor is enhanced in females, while males present higher rates of heterodimerization of Nk3R with the membrane androgen receptor

**Fig. 3:**
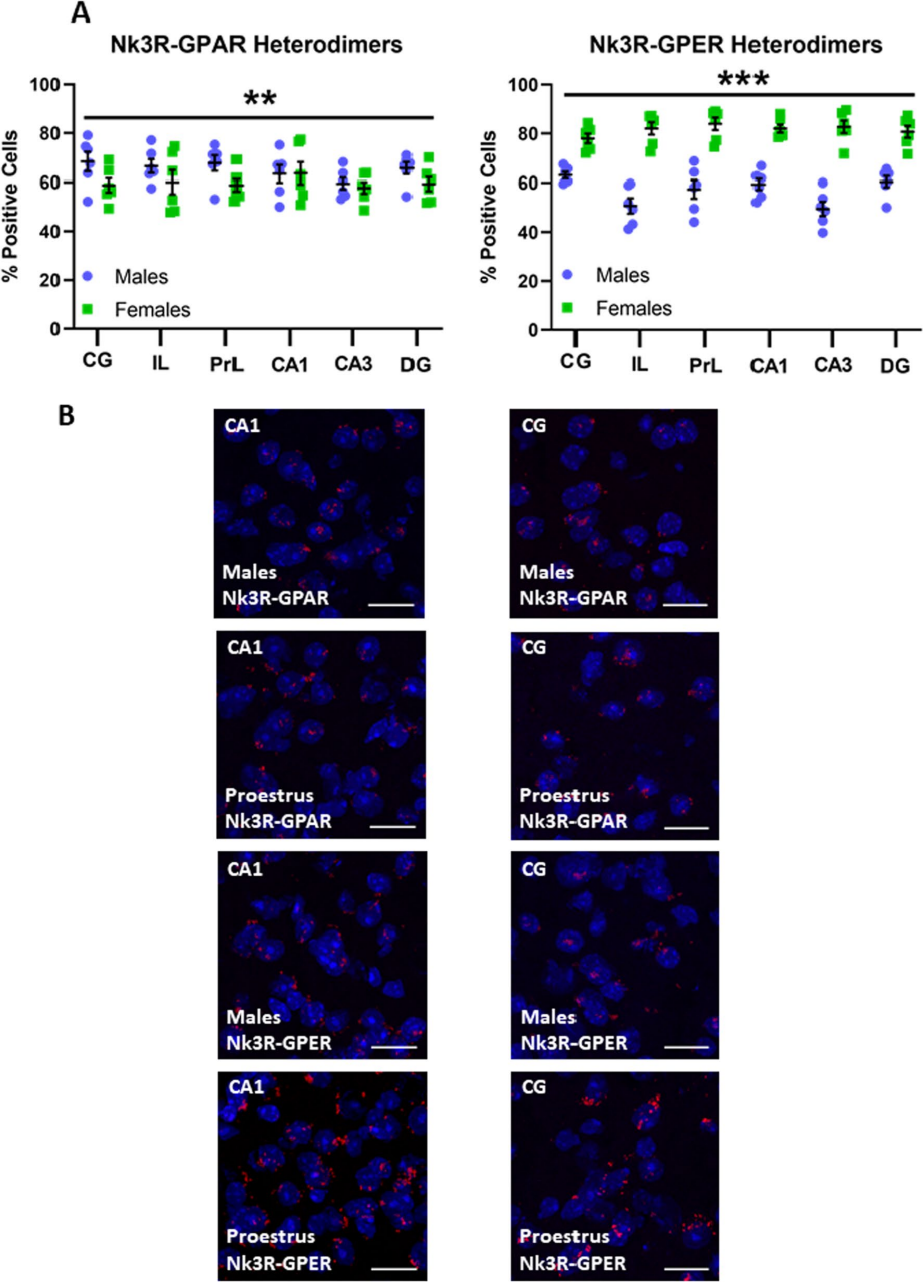
Heterodimerization of neurokinin 3 receptor with membrane sex hormones receptors. **A** Quantification of heterodimerization Nk3R with G-protein androgen receptor (GPAR) and G-protein estrogen receptor (GPER) in male and female mice during proestrus. *CG* cingulate cortex, *IL* infralimbic cortex, *PrL* prelimbic cortex, *CA1* field CA1 of hippocampus, *CA3* field CA3 of hippocampus, *DG* dentate gyrus. Data are mean ± SEM. Datasets were analyzed using two-way ANOVA. **p < 0.01, ***p < 0.001. **B** Representative confocal images of the previously reported quantification. Scale bar = 20 µm

We hypothesized a possible interaction between the Nk3R and the membrane GPER in females and with the membrane GPAR in male mice. Data showed significant interactions between Nk3R-ERα, Nk3-ERβ, and Nk3R-AR in proestrus females and males using in situ proximity ligand assay (PLA) in the DH and the mPFC. Given that estradiol and testosterone appear to be necessary for modulation of fear memories upon antagonism of the Nk3R in female and male mice, respectively [7], we aimed to test whether an activational effect of sex hormones could mediate the efficacy of Nk3R in the modulation of memory consolidation. To test that hypothesis, we assessed the presence of Nk3R–GPER and Nk3R–GPAR heterodimers in male and proestrus females through PLA in the DH and the mPFC. Results suggest significant interactions of area by sex [*F*(5,120) = 2.461, *p* = 0.037] and dimer by sex [*F*(1,120) = 159.754, *p* = 0.000] in the number of positive cells, while the interactions area by dimer ([*F*(5,120) = 0.383, *p* = 0.860]) and area by sex by dimer ([*F*(5,120) = 1.250, *p* = 0.290]) did not reach statistical significance (Fig. 3A, B, Tables 1, 2).

**Table 1:** Mean values and standard deviation (mean (SD)) for Nk3R–GPAR and Nk3R–GPER heterodimers in the mPFC and the DH

Area	GPAR		GPER	
	Males	Females	Males	Females
CA1	63.43 (8.92)	63.65 (11.09)	59.36 (5.56)	82.00 (4.01)
CA3	59.47 (5.80)	57.72 (5.79)	49.47 (7.04)	82.73 (6.38)
DG	65.73 (6.02)	59.26 (7.09)	60.48 (5.66)	80.70 (5.88)
CG	68.42 (9.60)	58.77 (7.08)	63.13 (3.21)	77.98 (4.78)
PrL	67.78 (7.72)	58.75 (6.14)	57.37 (9.17)	83.94 (6.31)
IL	66.57 (6.85)	60.07 (12.02)	50.69 (7.67)	82.07 (6.25)

The mean value was calculated based on the quantification of cells containing one or more heteromers versus total cells

*GPAR* G-protein androgen receptor, *GPER* G-protein estrogen receptor, *CG* cingulate cortex, *IL* infralimbic cortex, *PrL* prelimbic cortex, *CA1* field CA1 of hippocampus, *CA3* field CA3 of hippocampus, *DG* dentate gyrus

**Table 2:** Statistical tests for independent sample analyses

Between-subjects variables	Snedecor’s *F*		*p*-value
	*df*	Value	
Preference test			
Sex	1,24	0.057	0.813
Object	1,24	0.072	0.791
Sex × Object	1,24	0.072	0.791
Familiarization (5 mg/kg)			
Sex	1,56	0.000	1.000
Object	1,56	0.031	0.860
Treatment	1,56	0.000	1.000
Sex × Object	1,56	0.359	0.552
Sex × Treatment	1,56	0.000	1.000
Object × Treatment	1,56	1.128	0.293
Sex × Object × Treatment	1,56	0.006	0.939
NOR test (5 mg/kg)			
Sex	1,28	1.748	0.197
Treatment	1,28	0.227	0.603
Sex × Treatment	1,28	0.002	0.965
Familiarization (10 mg/kg)			
Sex	1,5	0.000	1.000
Object	1,5	0.000	1.000
Treatment	1,5	0.103	0.749
Sex × Object	1,5	0.091	0.764
Sex × Treatment	1,5	0.000	1.000
Object × Treatment	1,5	1.638	0.206
Sex × Object × Treatment	1,5	2.651	0.110
NOR test (10 mg/kg)			
Sex	1,25	10.595	*0.003*
Treatment	1,25	0.022	0.884
Sex × Treatment	1,25	17.128	*0.000*
Treatment in males	1,25	9.496	*0.005*
Treatment in females	1,25	7.714	*0.010*
Nk3R expression			
Sex	1,43	7.770	0.008
Area	5,44	1.631	0.257
Sex × Area	5,44	0.768	0.758
ERα expression			
Sex	1,47	7.001	0.011
Area	5,47	1.789	0.133
Sex × Area	5,47	1.326	0.270
ERβ expression			
Sex	1,41	115.275	*0.000*
Area	5,41	28.389	*0.000*
Sex × Area	5,41	26.475	*0.000*
Sex in CA1	1,41	21.410	*0.000*
Sex in CA3	1,41	0.001	0.973
Sex in DG	1,41	98.405	*0.000*
Sex in CG	1,41	105.908	*0.000*
Sex in IL	1,41	0.000	0.985
Sex in PrL	1,41	0.029	0.866
AR expression			
Sex	1,42	1.388	0.245
Area	5,42	9.144	*0.000*
Sex × Area	5,42	0.475	0.793
CG vs rest of areas	Bonferroni correction		*0.000*
Nk3R–GPAR positive cells			
Sex	1,60	8.402	*0.005*
Area	5,60	0.688	0.635
Sex × Area	5,60	0.717	0.613
Nk3R–GPER positive cells			
Sex	1,60	290.103	*0.000*
Area	5,60	1.609	0.171
Sex × Area	5,60	3.810	*0.005*
Sex in CA1	1,10	0.001	0.972
Sex in CA3	1,10	0.274	0.612
Sex in DG	1,10	2.906	0.119
Sex in CG	1,10	3.929	0.076
Sex in IL	1,10	3.929	0.076
Sex in PrL	1,10	5.033	*0.049*

Two or three-way ANOVAs were performed to assess statistical significance for independent sample analyses. Significant main effects and interactions between main effects are indicated in italic. Bonferroni post hoc analysis was used to assess significance between categories of a significant multiple-category main effect

Interaction between main effects area by sex and area by dimer describes an increased proportion of heterodimerized Nk3R with GPER in proestrus females compared to males in all areas examined ([*F*(1,120) = 213.664, *p* = 0.000]), with an increased proportion of heterodimerized Nk3R with GPAR in all assessed areas in male mice compared to proestrus female mice ([*F*(1,120) = 10.616, *p* = 0.001]). Differences in the number of heterodimers describe increased number of heterodimers in proestrus females compared to males in the DH in CA1 ([*F*(1,120) = 15.100, *p* = 0.000]), CA3 ([*F*(1,120) = 28.687, *p* = 0.000]) and the PrL ([*F*(1,120) = 8.884, *p* = 0.003]) and IL ([*F*(1,120) = 17.899, *p* = 0.000]), but not in the CG ([*F*(1,120) = 0.779, *p* = 0.379]) (Fig. 3A, B, Tables 1, 2), probably because of the higher rate of heterodimerization with GPER in all areas examined.

## Discussion

**Fig. 4:**
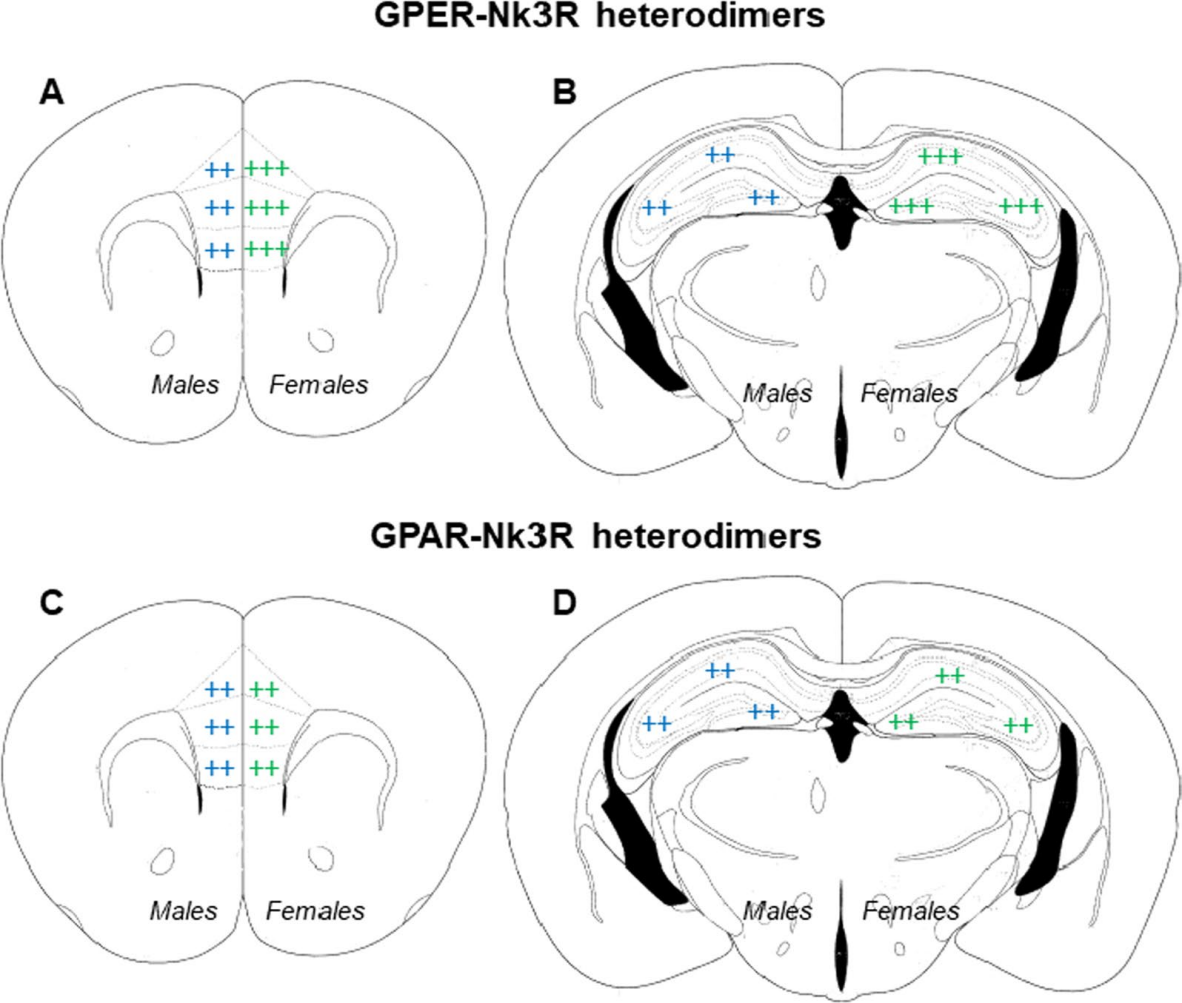
Schematic representation of Nk3R–GPER and Nk3R–GPAR in the dorsal hippocampus and the medial prefrontal cortex. Males are represented in blue on the left and females in green on the right. The percentage of positive cells is represented with the sign +. ++ indicates a medium expression (25–75%) and +++ indicates a high proportion (< 75%)

This study shows for the first time that the systemic blockade of the Nk3R reduces novel object recognition memory consolidation in male mice and enhances this same process in female mice that present high levels of circulating sex hormones. The Nk3R antagonist osanetant is a good candidate to study memory consolidation since it presents high blood–brain barrier penetration, a half-life of ~ 2 h [30], and a high clearance [31]. Notwithstanding, since the Nk3R is a GPCR, the effect of its blockade remains in the brain until phosphatases, kinases, and other metabolite levels return to the baseline. We measured the expression of the Nk3R and sex hormone receptors in the mPFC–hippocampal circuit that underlie the formation of novel object recognition memories. We also uncovered potential new protein interaction mechanisms between the Nk3R and membrane receptors for sex hormones (GPER and GPAR), in male and female mice, that may lead to opposite-sex effects of Nk3R targeted drugs on memory consolidation (Fig. 4).

### Sex differences in object recognition

Sex differences in recognition memory performance have been previously studied, although it is still controversial. While some authors report better performance for males in the NOR test [32], others report an outperformance of female mice [33]. This better performance of females has been hypothetically related to better object discrimination compared to male mice, as shown in other work [34]. In contrast, male mice have been shown to present a better discrimination index when the novel object resembles the object that they have been previously familiarized with [34]. Our study does not show a sex-dependent performance on the NOR test, nor in the preference test performed before the recognition test, suggesting that both objects used as novel and old were sufficiently different to produce a similar discrimination rate between sexes.

### Nk3R opposite-sex regulation of memory consolidation

The Nk3R has been previously linked to the performance of male subjects in the object recognition test. The administration of the Nk3R agonist Senktide enhances performance in the NOR test in male Wistar rats [9]. At a molecular level, our previous studies in fear memory show that the Tac2 pathway modulations in the central amygdala (CeA) reduce the activity of the Akt/GSK3β/β-Catenin in males and increases the activity of this molecular route in females leading to opposite-sex effects in memory [6, 7]. Our present study suggests that Nk3R antagonism may modulate in a similar manner recognition memory in both sexes with potential similarities in the mechanisms that mediate both fear and recognition memory. Increased activity of GSK3β prevents the phosphorylation of β-Catenin and, therefore, its role in the consolidation of memories. In the same line, increased activity of GSK3β has been shown to impair novel object recognition, as well as mood regulation and hippocampal cell proliferation [35]. Further, activation of the Akt/GSK3β pathway in the presence of estradiol has been shown to increase object recognition [22]. Taking all these data into account, we hypothesize a possible confluence in the mechanisms of memory consolidation after Nk3R antagonism, with the GSK3β downstream target β-Catenin modulating the formation of long-lasting memories.

In previous works [6, 7], we demonstrated that a single dose of 5 mg/kg reduces fear memory consolidation in males and increases it in females. In contrast, this dose was not effective in modulating recognition memory consolidation, but a 10 mg/kg dose reproduced this effect in the NOR task. As commented before, the neural networks supporting consolidation for fear memories and recognition memories remain similar between humans and rodents and differ consistently. Consolidation of cued-fear memories typically requires molecular and systemic changes in the amygdala and extra-amygdalar complexes [36, 37]. In contrast, consolidation of recognition memory has been widely associated with the hippocampus and its connections with the mPFC [2, 22, 38]. Each of these brain regions presents a unique organization and information flow, which may vary its sensitivity to osanetant modulation of synaptic and systemic consolidation processes.

Previous work has demonstrated that senktide, a potent Nk3R agonist, increases memory consolidation in male rats [9]. Also, senktide reverted the scopolamine-induced memory impairment, suggesting that the muscarinic cholinergic receptors mediate the memory enhancement of senktide [39]. Senktide has also been shown to increase acetylcholine release in the mPFC and the DH [40, 41]. A tentative explanation of the observed behavioral outcome is a decrease of ACh release in males and an increase in females, after osanetant administration. Notwithstanding, NkB also interacts with the dopaminergic system regulating dopamine release in the striatum in healthy and pathological individuals [42]. We suggest that further research should evaluate ACh release in the mentioned areas as well as the behavioral outcome of site-directed manipulations of Nk3R receptors.

### Sex hormones interaction with Nk3R on recognition memory consolidation

Estradiol has been shown in female mice to increase recognition memory consolidation by enhancing the activity of the Akt/GSK3β pathway in the DH [22]. Interestingly, wild-type mice, but not ERβ-KO, increased their performance on the object recognition task when treated with estradiol [43], suggesting a role for ERβ in the modulation of the enhancement effect of estradiol in object recognition memory consolidation. Our study shows that ERβ is more expressed in the CA1 and DG of the DH and the CG of the mPFC in females compared to males, suggesting possible target areas of estradiol in the enhancement of recognition memory consolidation, especially in those that represent the direct output of the DH. Notwithstanding, ERα is also present in the DH and mPFC of female mice although its expression is lower expressed than in males. Data in female Swiss mice have also highlighted the role of hippocampal ERα in enhancing memory consolidation [44].

Our study demonstrates a high degree of heterodimerization of the Nk3R with GPER in the DH and the mPFC, finding that around 80% of Nk3R found in these areas present heterodimerization with GPER in female mice (Fig. 4A, B). With these data, we hypothesize that estradiol exerts an activating function over the GPER that allows the inhibition of the Nk3R to increase intracellular cascades responsible for an increase in memory consolidation. Although not at a similar level, approximately 60% of Nk3R in the DH and the mPFC presents heterodimerization with membrane GPAR (Fig. 4C, D). Previous experiments have demonstrated that the memory consolidation effects observed after the administration of osanetant are dependent on the sexual maturity of the mouse [7], either male or female. Interestingly, given the involvement of sex hormones in the regulation of Nk3R activity on memory formation [7], estrogen GPCR has recently been shown to modulate hippocampal-dependent memory and long-term potentiation [45]. Although the Nk3R presents a significant number of heterodimers with AR in males and proestrus females, the Nk3R–GPER heterodimer appears quite more abundant than the rest, pointing to a possible explanation for the increased efficacy of Nk3R during high estradiol levels. A tentative hypothesis for this phenomenon is that the high degree of heterodimerization of Nk3R with sex hormones receptors, with new and unique pharmacological and functional properties different from those of its components [24, 46, 47], would produce a sex-specific activation of the heterodimer in the presence of sex hormones, allowing the antagonism to modulate memory consolidation in a sex-opposite way. It would be interesting for future studies to follow up our PLA analyses with co-immunoprecipitation to further understand protein–protein interactions of the Nk3R, ERα, ERβ, and AR. Additionally, an important question that could be further addressed is whether this results in heterodimerization of receptors that might interact with drug treatments, experience, or during the normal hormonal cycling or life-span in male and female mice. Further, we would like to highlight that our treatments take place 30 min after the familiarization phase of the NOR task, therefore, avoiding a possible effect of the drug in the test phase. Notwithstanding, we believe that the consolidation effect observed is affecting the storage of memories and therefore, retrieval. Further, we would also highlight that during the retrieval phase osanetant is no longer present in the animal’s body, so we could isolate the effect of the drug in the consolidation phase. Notwithstanding, it will be quite interesting to manipulate Tac2/Nk3R before or during retrieval to have a more complete knowledge of the phenomenon explained here.

### Perspectives and significance

This study demonstrates for the first time a sex-opposite effect of an Nk3R antagonist on recognition memory consolidation, together with a high degree of heterodimerization between Nk3R and membrane sex hormone receptors. Osanetant is a safe and well-tolerated drug in humans [10], an important finding that may lead to a rapid translation of these results into the clinic. Osanetant has not been approved yet for the treatment of mental disorders in humans, but there are clinical trials currently studying this possibility.

Previous research has shown that Nk3R is expressed in Tac2 neurons in the centromedial amygdala (CeM) [4]. As previously demonstrated [7], these neurons in the CeM are GABAergic neurons, concordantly with the anatomical data available on the distribution of GABA and glutamate in the amygdala. Notwithstanding, few articles address the colocalization of Tac2 with other neuropeptidergic systems. One example of this colocalization is the Tac2-Kisspeptin neurons in the hypothalamus [48]. Following this, Kisspeptin is not one of the representative neuropeptides found in the CeM, suggesting that the colocalization with other neuropeptidergic systems may vary depending on the area. The specific expression of Tac2 and its colocalization in the medial prefrontal cortex and the dorsal hippocampus has not been addressed in this article and could be of interest for future research.

Interestingly, Nk3R pharmacological manipulations have aroused as a promising new tool for memory deficits through its interaction with the cholinergic system [40, 41]. Moreover, interactions of the Tac2 pathway and GABA in the extended amygdala modulate the activity of the lateral hypothalamic area [47]. All these findings highlight exciting possible novel pharmacological targets for the treatment of anterograde amnesic affection in women, suggesting a path for future research on personalized medicine that tests the possibilities of Nk3R antagonists in the treatment of memory loss in women. Moreover, the impaired memory consolidation of osanetant in males in the consolidation of both fear memory and episodic memory may be a tool for the treatment of aversive events with an emotional and episodic memory component.

## Data Availability

All data and materials are available from the corresponding author upon reasonable request.
